# Magnetic field tuning of an excitonic insulator between the weak and strong coupling regimes in quantum limit graphite

**DOI:** 10.1038/s41598-017-01693-5

**Published:** 2017-05-04

**Authors:** Z. Zhu, R. D. McDonald, A. Shekhter, B. J. Ramshaw, K. A. Modic, F. F. Balakirev, N. Harrison

**Affiliations:** 1 MS-E536, NHMFL, Los Alamos National Laboratory, Los Alamos, New Mexico, 87545 USA; 2 Wuhan National High Magnetic Field Center, School of Physics, Huazhong University of Science and Technology, 1037 Luoyu Road, 430074 Wuhan, China; 3 National High Magnetic Field Laboratory, Florida State University, 1800 E. Paul Dirac Dr., Tallahassee, Florida, 32310 USA; 4 Laboratory of Atomic and Solid State Physics, Cornell University, Ithaca, NY 14853 USA; 5 Max Planck Institute for Chemical Physics of Solids, Nöthnitzer Strape 40, Presden, 01187 Germany

## Abstract

The excitonic insulator phase has long been predicted to form in proximity to a band gap opening in the underlying band structure. The character of the pairing is conjectured to crossover from weak (BCS-like) to strong coupling (BEC-like) as the underlying band structure is tuned from the metallic to the insulating side of the gap opening. Here we report the high-magnetic field phase diagram of graphite to exhibit just such a crossover. By way of comprehensive angle-resolved magnetoresistance measurements, we demonstrate that the underlying band gap opening occurs inside the magnetic field-induced phase, paving the way for a systematic study of the BCS-BEC-like crossover by means of conventional condensed matter probes.

## Introduction

Half a century ago, Mott pointed out that tuning the carrier density of a semimetal towards zero produces an insulating state in which electrons and holes form bound pairs^1^. It was later argued that such pairing persists even if a semiconducting gap opens in the underlying band structure, giving rise to what has become known as the strong coupling limit of an ‘excitonic insulator’^2^. These ‘weak’ and ‘strong’ coupling extremes on either side of the band gap opening were subsequently proposed to be manifestations of the same excitonic state of electronic matter^3–7^. Studies of photo-excited excitons in semiconductors have provided indirect evidence that these two extremes are connected via a crossover^8–11^.

The hallmark of an excitonic insulator is the spontaneous formation of a broken symmetry phase in equilibrium that straddles both sides of a band gap opening in the underlying band structure^4, 5, 7^. On the weak coupling side, electrons and holes pair at the Fermi surface in direct analogy to electron-electron pairing in Bardeen-Schrieffer-Cooper (BCS) superconductors^7, 12^. On the strong coupling side, bound electron-hole pairs form across a semiconducting gap giving rise to an exciton gas which can subsequently condense. The symmetry broken by the ground state is expected to depend on the specifics of the band structure and can include a Bose-Einstein Condensate (BEC) of excitons^7^, a Wigner crystalline solid^5^ (i.e. a strong coupling variant of a spin- or charge-density wave) or a state with chiral symmetry breaking^13, 14^. Despite extensive experimental searches for a phase transition into an excitonic insulator phase bridging the weak and strong coupling regimes, only the weak coupling regime has thus far been reported^15, 16^.

Here we show the quantum limit of graphite^17–19^, by way of temperature and angle-resolved magnetoresistance measurements, to host an excitonic insulator phase that evolves continuously between the weak and strong coupling limits in equilibrium. We find that the maximum transition temperature *T*_EI_ ≈ 9.3 K of the excitonic phase is coincident with a band gap opening in the underlying electronic structure at *B*_0_ = 46 ± 1 T, which is evidenced above *T*_EI_ by a thermally broadened inflection point in the magnetoresistance. The overall asymmetry of the observed phase boundary around *B*_0_ resembles the original theoretical predictions of a magnetic field-tuned excitonic insulator phase^4, 5, 7, 20, 21^, suggesting a smooth crossover between the BCS and BEC regimes with increasing magnetic field^4, 5, 7^.

**Figure 1:**
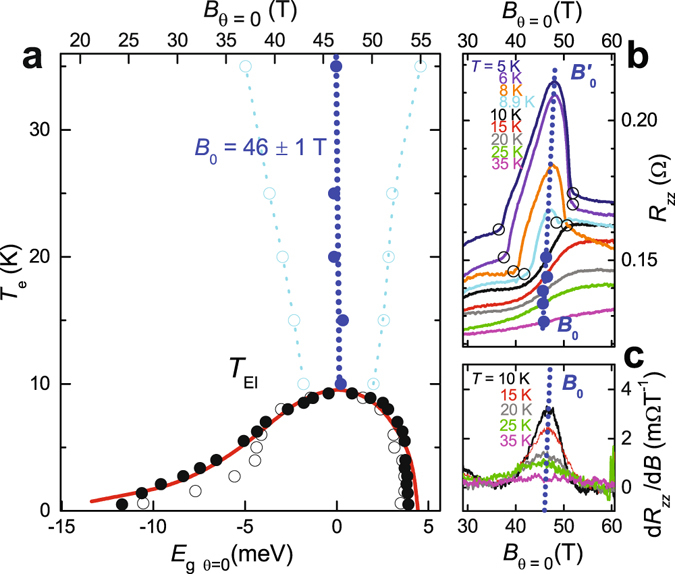
(**a**) Experimental phase boundary at *θ* = 0° (filled black circles) from *R*_*xx*_ (see Supplementary Information) and the onset of insulating behavior (open black circles) from *R*_*zz*_, plotted against *B* (top axis) and *E*_g_ according to Equation 1 with *E*_0_ = 24.4 meV (bottom axis). The red line indicates the mean field phase boundary of Abrikosov^20^. Cyan circles (and dotted line) indicate the half-maximum width of ∂*R*_*zz*_/∂*B*. (**b**) *R*_*zz*_ in the vicinity of the excitonic insulator phase at selected temperatures as indicated. (**c**) ∂*R*_*zz*_/∂*B* versus *B* for *T* > *T*_EI_. In all panels, filled blue circles indicate the point of inflection in *R*_*zz*_ at *T* > *T*_EI_, with a blue dotted line providing a guide to the eye. Note that *E*_g_ is only weakly *T*-dependent (see Supplementary Information).

**Figure 2:**
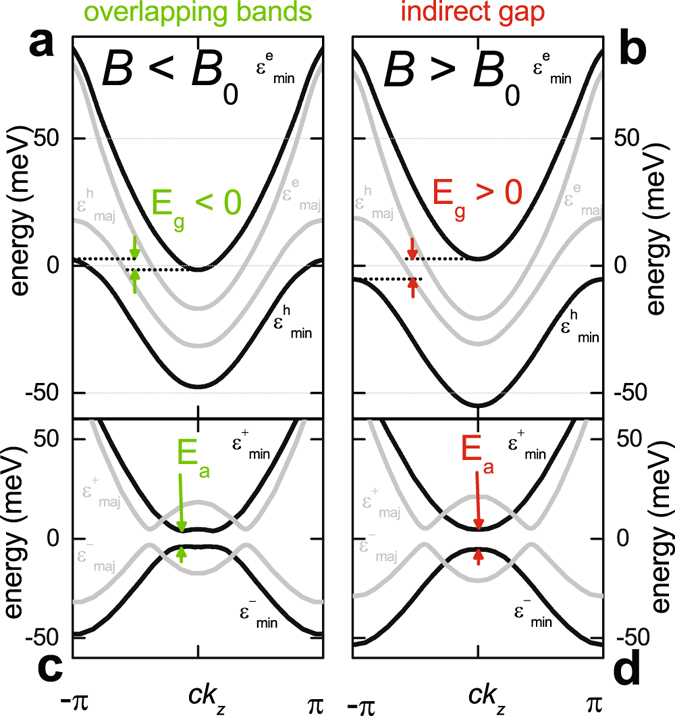
(**a**) Electronic dispersion of the Landau subbands according to ref. 19 at *B* < *B*_0_, giving rise to a small overlap (*E*_g_ < 0) between the minority-spin electron (${\varepsilon }_{{\rm{\min }}}^{{\rm{e}}}$) and hole (${\varepsilon }_{{\rm{\min }}}^{{\rm{h}}}$) Landau subbands (depicted in black). The majority-spin bands (${\varepsilon }_{{\rm{maj}}}^{{\rm{e}}}$) and (${\varepsilon }_{{\rm{maj}}}^{{\rm{h}}}$) are depicted in grey. (**b**) Electronic dispersion at *B* > *B*_0_, giving rise to a small gap (*E*_g_ > 0) between the minority-spin electron and hole bands. (**c**) Schematic dispersion for a spin-triplet excitonic insulator phase (a spin-density-wave for weak coupling that doubles the *c*-axis unit cell) for *E*_g_ < 0. The folded dispersion is calculated from the anticrossing of the translated bands with the exciton gap function Δ using ${\varepsilon }_{{\rm{\min }}\,,{\rm{maj}}}^{\pm }=\frac{1}{2}[{\varepsilon }_{{\rm{\min }}\,,{\rm{maj}}}^{{\rm{e}}}({k}_{z})+{\varepsilon }_{{\rm{\min }}\,,{\rm{maj}}}^{{\rm{h}}}({k}_{z}+{Q}_{z})]\pm \sqrt{\frac{1}{4}{[{\varepsilon }_{{\rm{\min }},{\rm{maj}}}^{{\rm{e}}}({k}_{z})-{\varepsilon }_{{\rm{\min }},{\rm{maj}}}^{{\rm{h}}}({k}_{z}+{Q}_{z})]}^{2}+{{\rm{\Delta }}}^{2}}$. (**d**) Same as (**c**) but for *E*_g_ > 0.

The sharp phase transitions in quantum limit graphite above 20 T (see Fig. 1) have been the subject of numerous experimental studies^16, 22–25^. Our experimental phase boundary (solid black circles in Fig. 1a) is traced from both inter-plane (see Fig. 1b) and in-plane resistance data (see Supplementary Information). While the field-induced insulating behavior has been associated with the formation of a field-induced density-wave phase^19, 26–28^, the relationship of the density-wave phase to the opening of a band gap in the underlying electronic structure has remained undetermined. In the absence of a direct measurement of the underlying gap, it has been assumed from fixed angle studies performed thus far (i.e. *θ* = 0°)^16, 24, 25^ that a band gap opening coincides with the upper magnetic field phase boundary of the phase near ≈54 T^19^ (see Fig. 1a). Such an analysis has suggested that the entire magnetic field-induced phase lies on the weak coupling BCS side where Landau subbands always overlap (i.e. Fig. 2a)^26^ and where pairs are formed by connecting opposing momentum-states on the Fermi surface.

Rather than being coincident with the upper magnetic field boundary of the phase, we show here that the band gap opening in the underlying electronic structure lies in close proximity to the magnetic field at which the transition temperature is maximum, therefore exhibiting the signature characteristics of an excitonic insulator phase^4, 5, 7, 20^. Experimental evidence for the band gap opening at a magnetic field ≈46 T, substantially below the upper boundary of the field-induced insulating phase near 54 T, is provided by a point of inflection in the inter-plane electrical resistance *R*_*zz*_ at temperatures above *T*_EI_ (solid blue circles in Fig. 1). In the absence of ordering, the sudden emptying of electron and hole states upon opening of the band gap (*E*_g_) is expected to result in a discontinuity (i.e. a step) in the electrical resistivity in the limit of zero temperature^5^. When broadened by the Fermi-Dirac distribution at finite temperatures and by other factors such as fluctuations and a finite relaxation time, this becomes a point of inflection. The thermal evolution of the width of the peak in the derivative ∂*R*_*zz*_/∂*B* in Fig. 1c shows that the point of inflection becomes increasingly sharp and step-like on lowering the temperature towards *T*_EI_, making it consistent with a discontinuity at *B*_0_ in the underlying band structure at low temperatures. Importantly, no high temperature feature is seen to occur in *R*_*zz*_ at 54 T, where the band gap was previously assumed to open^16, 19, 24, 25^, suggesting its shifting to the lower magnetic field value of *B*_0_ ≈ 46 T by the effects of electronic correlations^19^.

**Figure 3:**
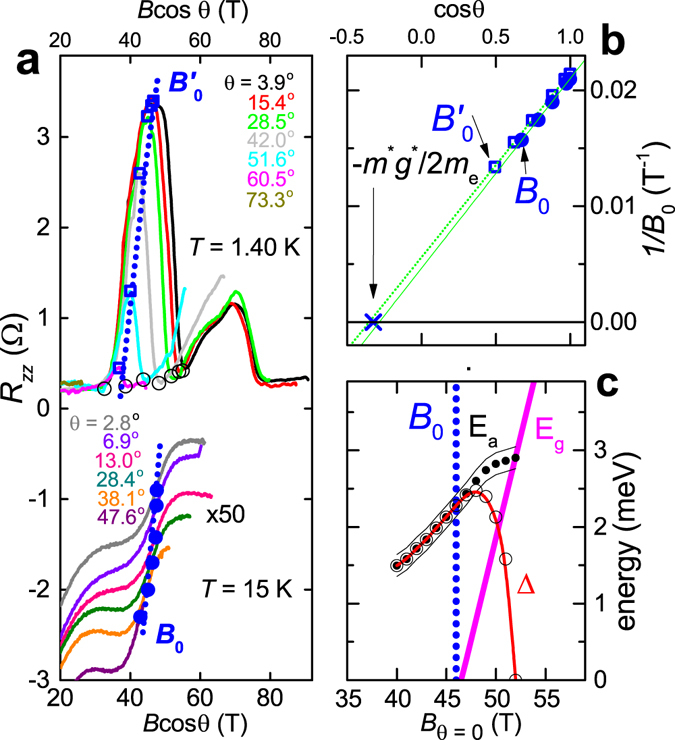
(**a**) *R*_*zz*_ at *T* = 1.40 K and *T* = 15 K at several angles *θ* as indicated (15 K curves shifted for clarity). Open black circles indicate the onset of insulating behavior (for *T* ≤ *T*_EI_). (**b**) Plots of 1/*B*_0_ and $1/{B}_{0}^{^{\prime} }$ versus cos *θ*, yielding ${m}^{\ast }{g}^{\ast }$ estimates from the intercept of fits to Equation 1 (green line and dotted lines respectively). The fields at which the inflection point occurs are obtained from the peak in the derivative ∂*R*_*zz*_/∂*B* (see Supplementary Information). Blue dotted lines are a guide to the eye. (**c**) A comparison of the field dependence of the energy gap *E*_a_ (circles with thin black lines indicating the error bars) estimated from thermally activated _*zz*_^25^ (see Supplementary Information) with the band gap *E*_g_ (magenta). Open circles connected by a red curve indicate the gap function estimated using ${\rm{\Delta }}=\sqrt{{E}_{{\rm{a}}}^{2}-{E}_{{\rm{g}}}^{2}}$.

It should be noted that once density-wave ordering sets in at temperatures below *T*_EI_ (onset indicated by filled circles in Fig. 1), the development of insulating behavior in *R*_*zz*_ (onset indicated by open circles) implies that the electronic structure must become almost entirely gapped. We find the maximum in *R*_*zz*_ within the insulating phase to be located at a very similar field ${B}_{0}^{^{\prime} }\approx 47\,{\rm{T}}$ to *B*_0_ (see Figs 1 and 3a and Supplementary Information). One possible explanation for the insulating behavior below *T*_EI_ is provided by the density-wave excitonic insulator scenario depicted in Fig. 2c,d, whereby the density-wave is primarily hosted by the minority-spin states but also induces a secondary gap to open on the majority-spin Fermi surface (the electronic density-of-states of the majority-spin Fermi surface at 46 T being significantly smaller than that of the minority-spin Fermi surface).

The key experimental evidence for the band gap opening between minority-spin electron and hole bands of graphite (shown in Fig. 2a,b) is provided by our angle-resolved measurements shown in Fig. 3. Because the spin and orbital contributions to *E*_g_ have differing dependences on the orientation of the magnetic field in layered materials, angle-resolved measurements (see Fig. 3) enable the spin and orbital contributions to be selectively tuned. The inflection in *R*_*zz*_ at *B*_0_ ≈ 46 T and the maximum in *R*_*zz*_ within the excitonic insulator phase at ${B}_{0}^{^{\prime} }\approx 47\,{\rm{T}}$ in Figs 1b and 3a are both observed to shift in field on increasing the polar angle *θ* between the magnetic field and the crystalline *c*-axis. Their angle-dependences match the behavior expected for the opening of this band gap

1 $${E}_{{\rm{g}}}=\frac{\hslash e}{{m}^{\ast }}B\,\cos \,\theta +{g}^{\ast }{\mu }_{{\rm{B}}}B-{E}_{0}$$

between the lowest Landau levels of minority-spin electrons and holes due to the competition between quasi-two-dimensional Landau quantization and isotropic Zeeman splitting. Here, *E*_g_ is positive for *B* > *B*_0_ and negative (corresponding to a band overlap) for *B* < *B*_0_ (see schematic in Fig. 2a,b). The first term on the right-hand-side (in which ${m}^{\ast }$ is an effective mass that characterizes the splitting between the lowest electron and hole Landau levels^19^) results from orbital quantization within the two-dimensional honeycomb layers, the second term (in which ${g}^{\ast }$ is the effective *g*-factor, which is approximately isotropic in graphite, and *μ*_B_ is the Bohr magneton) results from the Zeeman coupling of the magnetic field to the electron spin while the third (*E*_0_) is a constant relating to the inter-plane electronic band structure of graphite. (Equation (1) is strictly valid only at *B* ≈ *B*_0_ where a singularity in the minority spin electronic density-of-states causes it to dominate the total density-of-states^19^, and at $\theta \mathop{ < }\limits_{ \tilde {}}60^\circ$ where the effect of the interlayer dispersion on the orbital quantization is negligible^29^. When the magnetic field is reduced to *B* cos *θ* ≈ 25 T (where the onset of the field-induced phase occurs), the electronic density-of-states of the minority and majority spin components are similar^19^ causing the effect of the Zeeman term to be negligible. In this limit, the field induced spin-density wave (or charge density-wave state) is BCS-like^26^ and its onset depends only on the total electronic density-of-states, which depends on *B* cos *θ* to leading order^23^).

Defining *B*_0_ as the field at which the band gap opens (i.e. *E*_g_ = 0), Equation 1 produces a linear dependence of 1/*B*_0_ on cos *θ*, with an offset of −$({m}^{\ast }/{m}_{{\rm{e}}}){g}^{\ast }/2$. On plotting the 1/*B*_0_ data versus cos *θ* in Fig. 3b, the intercept of the fitted solid green line yields $({m}^{\ast }/{m}_{{\rm{e}}}){g}^{\ast }/2\approx 0.284$ (where *m*_e_ is the free electron mass). The near coincidence of ${B}_{0}^{^{\prime} }$ below *T*_EI_ with *B*_0_ above *T*_EI_ suggests that it can be used to provide an independent estimate of $({m}^{\ast }/{m}_{{\rm{e}}}){g}^{\ast }/2$ (see Supplementary Information). On plotting the $1/{B}_{0}^{^{\prime} }$ data versus cos *θ* in Fig. 3b, the intercept of the fitted dotted green line yields $({m}^{\ast }/{m}_{{\rm{e}}}){g}^{\ast }/2\approx 0.352$. The average 0.32 ± 0.03 of the two intercepts (indicated by an X symbol in Fig. 3b) is similar to the value ≈0.37 expected from the known parameters of graphite (${g}^{\ast }=2.5$ ^30^ and $({m}^{\ast }/{m}_{{\rm{e}}})=0.3$ ^18, 19^). (This effective mass parameter corresponds to the magnetic field-dependence of the energy difference between *n* = 0 (electron) and *n* = −1 (hole) Landau levels^19^, and is larger than the effective mass (≈0.05 *m*_e_) of the electron and hole pockets).

Our measurements identify the band gap opening in the underlying electronic structure to coincide with the maximum *T*_EI_ of the asymmetric excitonic phase boundary (black circles in Fig. 1a), resembling theoretical predictions made in the high magnetic field limit^20^ (red line). The physical situation can therefore be described as follows: electron-hole pairing for *B* < *B*_0_ occurs at the Fermi surface in momentum-space in accordance with a BCS-like transition into a weakly coupled spin- or charge-density wave phase^26–28^ (schematic in Fig. 2c). Such behavior has been confirmed experimentally by the observation of an exponential increase in the transition temperature with increasing magnetic field^31^. At *B* ≈ *B*_0_, however, singularities in the electronic density-of-states at the top of the minority-spin hole band and bottom of the minority-spin electron band coincide with the chemical potential, causing strongly bound minority-spin pairs to greatly outnumber weakly bound majority-spin pairs and therefore dominate the thermodynamics. When *B* > *B*_0_, the minority-spin pairing takes place across a band gap, thereby becoming local excitonic in nature^2^ (schematic in Fig. 2d). Pairing across a band gap is predicted to give rise to an increasingly dilute density of excitons as the magnetic field is increased^4, 5, 7, 20^. The exciton gap function, Δ, is expected to approach zero at the upper extremity of the phase (near ≈ 54 T in Fig. 1a). The total minority-spin energy gap, which will determine the thermally activated transport properties of such a correlated electron state, is given by the band gap and correlation gap added in quadrature ${E}_{{\rm{a}}}=\sqrt{{E}_{{\rm{g}}}^{2}+{{\rm{\Delta }}}^{2}}$. This gap becomes comparable to the band gap *E*_g_ when the exciton density vanishes^4, 5, 7, 20^. Such behavior is demonstrated in Fig. 3c by the evolution of an activation gap within the excitonic insulator phase, obtained from Arrhenius plots of *R*_*zz*_^25^ (see Supplementary Information), that continues to grow in the region *B* > *B*_0_, and then intersects with *E*_g_ on approaching the upper phase boundary. The point of intersection provides a lower bound estimate of ≈3 meV for the exciton binding potential energy, which is expected to be similar to the value of Δ at the peak transition temperature^4, 5, 7^. On estimating Δ from *E*_g_ given by Equation (1) and experimental *E*_a_ data in Fig. 3c, we find Δ to peak near *B*_0_ and then collapse rapidly to zero at 52 T, which is consistent with a scenario in which an Excitonic phase forms around a band gap opening^4, 5, 7, 20^.

**Figure 4:**
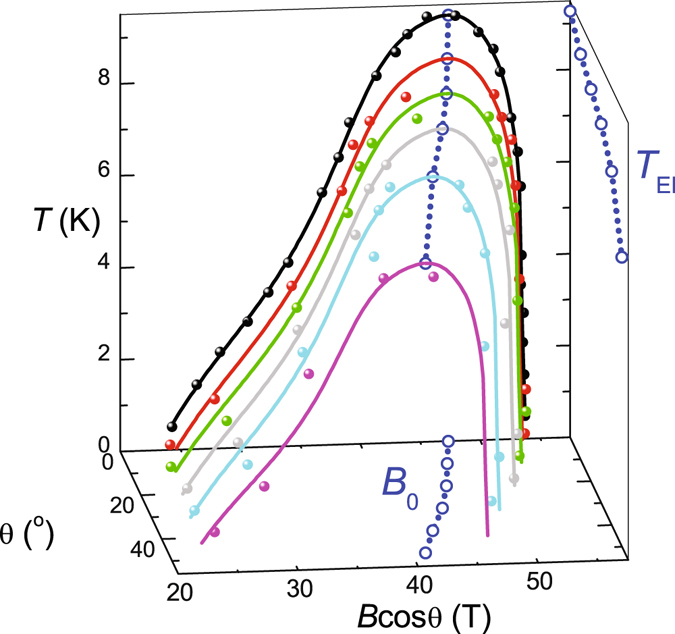
Excitonic phase boundary versus *θ*, where solid lines represent a spline fit to the phase boundary at *θ* = 0 which for *θ* > 0 has been rescaled as a guide as a guide to the eye. Blue circles connected by dotted lines represent the interpolated optimal *T*_EI_ at each *B*_0_, which has further been projected onto the *T* − *θ* and *B* cos *θ* − *θ* planes.

The stability of the excitonic insulator phase centered around *B*_0_ depends on the effective strength of the interactions determining the binding energy. The combination of anisotropic orbital and isotropic Zeeman contributions to *E*_g_ (as defined by Equation 1) shifts the opening of the band gap and hence the optimal transition temperature of the exitonic insulator to lower values of the component of magnetic field perpendicular to the planes, *B*_0_ cos *θ*, as *θ* is increased (Figs 3 and 4). The reduced optimal transition temperature of the excitonic insulator phase and its reduced extent in *B* cos *θ* at higher angles suggest that the maximum pairing strength at *E*_g_ = 0 is weakened at higher angles by the reduction in Landau level degeneracy, caused by the singularity in the density-of-states being shifted to lower values of *B* cos *θ*. The angle-dependent measurements hence provide an experimental means of tuning the pairing strength in a condensed matter system, independent of the electron gas density, analogous to that achieved in cold atomic gases^32^.

While the nature of the broken symmetry in quantum-limit graphite has remained an open question^16, 24–28^, our observation of the maximum transition temperature at the field *B*_0_ implies that the broken symmetry accompanying its formation bridges opposing limits of the phase diagram in which excitons are strongly and weakly bound. Beyond the proposed formation of a density-wave in the low-field, weak-coupling limit, the possibilities for the broken symmetry in the excitonic phase include a Bose-Einstein condensate of excitons^7^, a Wigner crystalline^5^ or supersolid^33^ state of excitons, or a state with chiral symmetry breaking^13, 14^. One way of forming a reconstructed electronic dispersion^19^ typical of an excitonic phase^4, 5, 20^ is a spin-ordered phase with an inter-plane component to the ordering vector of *Q*_*z*_ = *π*/*c* (shown schematically in Fig. 2c,d to couple electrons and holes of opposite spin). This has the attractive property of producing broken translational symmetry along the *c*-axis, as expected for a crystalline exciton phase^5^, while leaving the in-plane mobility of the electrons and holes intact and open to the possibility of superfluid^7^ or supersolid behavior^33^. This same *Q*_*z*_ vector also nests the majority-spin bands, which must ultimately be important for increasing the resistivity of the high field state.

Our observation of an ordered excitonic phase nucleating around the opening of a band gap, suggests that graphite is an attractive material for investigating exotic ordered states in ultra-low density electronic systems^17, 19, 34^ with poorly screened coulomb interactions^1^. The nature of the broken symmetry in the excitonic insulator phase and whether the onset of the insulating phase precedes or is coincident with it remains an open question. In particular, there exists a second field-induced phase at higher magnetic fields centered on ≈70 T, as reported by Fauqué *et al*.^25^, raising the possibility that this is a second excitonic insulator phase involving only the majority-spin carriers (the upper phase between ≈55 and 75 T also being evident in Fig. 3c). The similarity in shape of the second magnetic field-induced phase to that at low fields suggest that it may be centered around a band gap opening between the majority-spin Landau subbands at ≈70 T. Further measurements of *R*_*zz*_ at higher temperatures around 70 T and angle-resolved measurements made at higher magnetic fields ought to reveal whether or not a second majority-spin band gap opening occurs at this field.

## Electronic supplementary material

Supplementary Information
