# Supplementary material for: Magnetic field tuning of an excitonic insulator between the weak and strong coupling regimes in quantum limit graphite
Source: Sci Rep. 2017 May 4;7:1733. doi: 10.1038/s41598-017-01693-5 (PMC5431932; doi:10.1038/s41598-017-01693-5)
Supplement: Supplementary file 1 — Supplementary Information [file 41598_2017_1693_MOESM1_ESM.pdf]

Supplementary Information for "Magnetic field tuning of an excitonic insulator between the weak and strong coupling regimes in quantum limit graphite"

Zengwei Zhu,[1,2,*] Ross. D. McDonald,[1] Arkady Shekhter[1,3] Brad Ramshaw,[1] Kimberly A. Modic,[1] Fedor Balakirev[1] and Neil Harrison[1]

[1]*MS-E536, NHMFL, Los Alamos National Laboratory, Los Alamos, New Mexico 87545*

[2]*Wuhan National High Magnetic Field Center, School of Physics, Huazhong University of Science and Technology, 1037 Luoyu Road, 430074 Wuhan, China*

[3]*National High Magnetic Field Laboratory, Florida State University, 1800 E. Paul Dirac Dr., Tallahassee, Florida 32310*

(Dated: July 23, 2016)

I. SAMPLE PREPARATION AND MEASUREMENT

Angle- and temperature-dependent measurements of R_{xx} and R_{zz} were performed on natural graphite provided by Asbury Carbons, with the samples immersed in liquid or gaseous ^{3}He or ^{4}He throughout the experiment. Angle-dependent R_{xx} and R_{zz} measurements were performed on different samples using an in-situ sample rotator, while magnetic fields extending either to 65 T or 90 T were provided by the National High Magnetic Field Laboratory. The samples were platelets of thickness ≈ 30 μm and in-plane size ≈ 1 mm $\times$ 0.5 mm. For the R_{zz} measurements, the negative and positive polarity current and voltage leads were attached to opposite sides of the samples. For the R_{xx} and R_{xy} measurements, the leads were attached close to the edges on the same side of the sample.

The step in R_{zz} at $T > T_{\rm EI}$ was observed in multiple samples, with different samples being used for the R_{zz} measurements performed as a function of angle (Fig. 3 of the main text) and as a function of temperature (Fig. 1 of the main text). We were able to rule out the presence of a significant Hall contribution to R_{zz} measurements by reversing the direction of the applied magnetic field in-situ. We note that while our experimental phase boundaries are very similar to those on Refs. [2, 3], no systematic high magnetic field measurements of R_{zz} versus T have previously been made at $T > T_{\rm EI}$, with the exception of a single curve at $T = 10$ K in Ref. [3].

II. PHASE BOUNDARY

The phase boundary is discernible from kinks in the magnetoresistance measurements (see Figs. 1), with the transitions being visible in both R_{xx} (see Fig. 2) and R_{zz} (see Figs. 3 and 4). As the field is increased, the highly resistive insulating behavior in R_{zz} is achieved at a slightly higher magnetic field than the initial phase transition in R_{xx} and R_{zz}. Since insulating behavior in R_{zz} can only occur when the entire Fermi surface is gapped, we attribute the occurrence of insulating behavior in R_{zz} at the slightly higher magnetic field to the onset of complete gapping of the entire Fermi surface.

In the case of R_{xx} (see Fig. 2), we have adopted the notation of Yaguchi and Singleton for labeling the features in the magnetotransport [2]. We have assumed α and α' to bound the excitonic insulator phase (which have been replotted in Fig. 2A of the main text) Meanwhile,

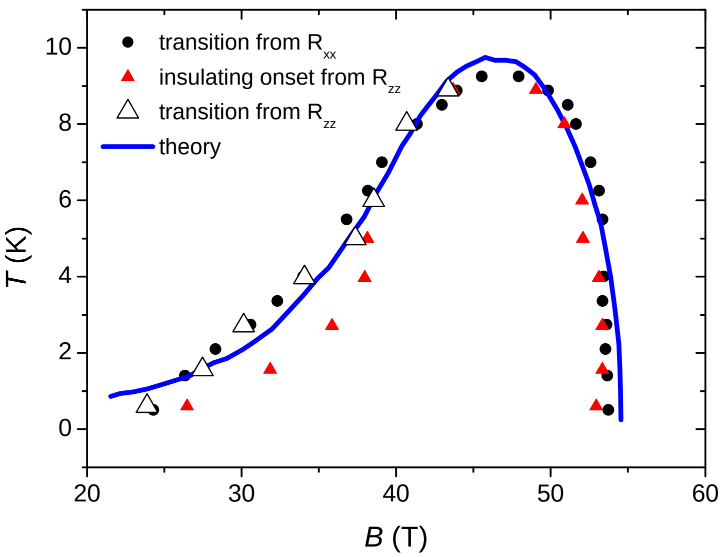

FIG. 1. The phase diagram obtained from kinks in R_{xx} (black circles) and R_{zz} (open black triangles), together with the onset of insulating behavior (red triangles). The blue line is the theoretical model of Abrikosov [1] arbitrarily rescaled.

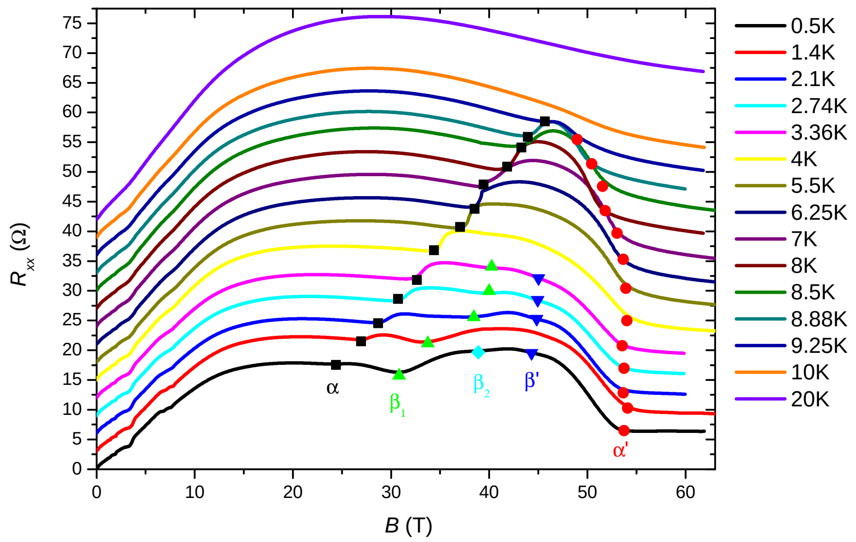

FIG. 2. R_{xx} with different temperatures depicted in different colors, as indicated. The curves have been shifted for clarity.

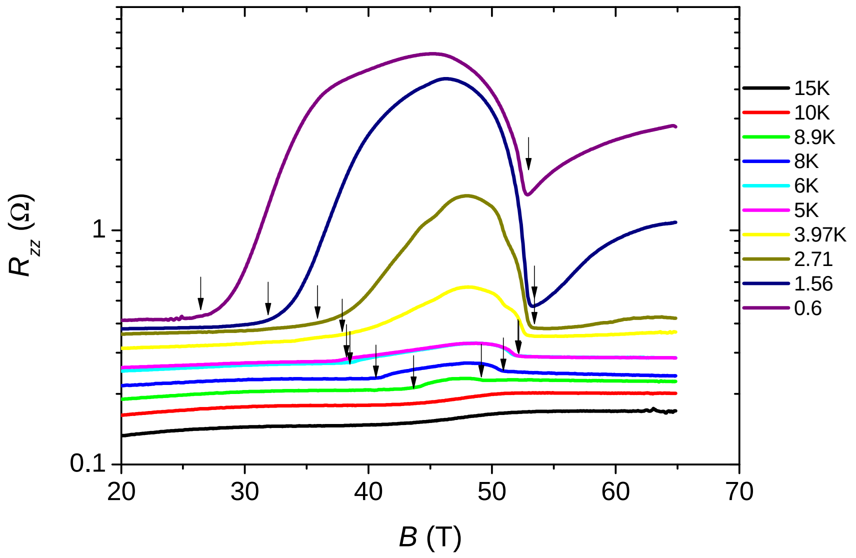

FIG. 3. R_{zz} with different temperatures depicted in different colors, as indicated. The curves have been shifted for clarity.

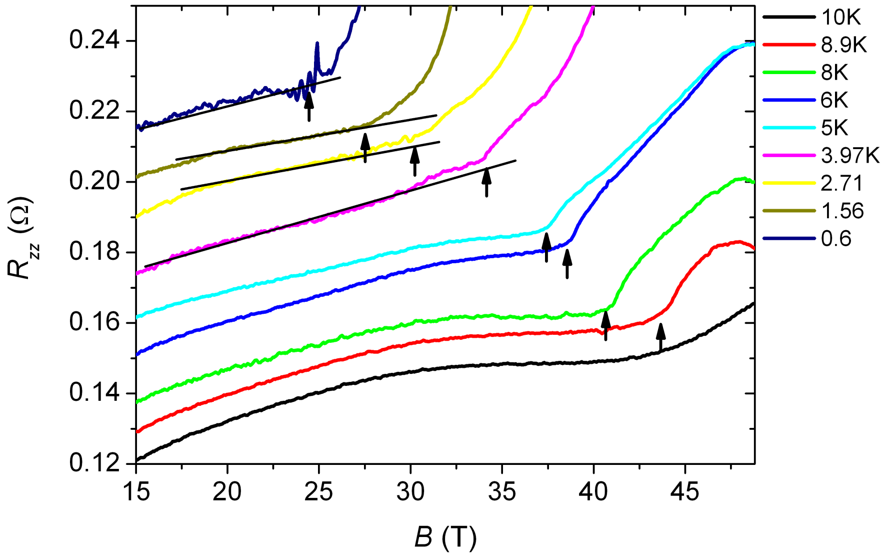

FIG. 4. Expanded view of R_{zz} at different temperatures in different colors, as indicated, showing the phase transition prior to the onset of insulating behavior (at high magnetic fields). The curves have been shifted for clarity.

β_1 corresponds to the onset of more strongly insulating behavior in R_{zz}. β_2 and β' have unknown origin, although the latter is close to the field B_0 at which a gap opens in the non-interacting electronic structure. At temperatures $T > T_{\rm EI}$, there is an inflection in R_{xx} close to B_0, although it appears to be much weaker than that in R_{zz}.

III. INSULATING BEHAVIOR

Since the interlayer resisivity ρzz is given simply by the reciprocal $\rho_{zz} = 1/\sigma_{zz}$ of the inter-plane conductivity, the observation of insulating behavior in R_{zz} within the excitonic insulator phase (in which the resistivity and resistance increases on decreasing the temperature) is consistent with a completely gapped state in which the excitons carry no electrical current perpendicular to the layers.

The diagonal (ρ_{xx}) and off-diagonal (ρ_{xy}) in-plane resistivity components, meanwhile, are given by

$$\begin{pmatrix} \rho_{xx} & -\rho_{xy} \\ \rho_{xy} & \rho_{yy} \end{pmatrix} = \begin{pmatrix} \sigma_{xx} & -\sigma_{xy} \\ \sigma_{xy} & \sigma_{yy} \end{pmatrix}^{-1}.$$

In this case, the presence of a Hall conductivity σ_{xy} that is larger than σ_{xx} could inhibit in-plane insulating behavior. We instead find that $R_{xx} \gg |R_{xy}|$ (see Fig. 5, indicating that $\sigma_{xx} \gg |\sigma_{xy}|$) and that the transition into the excitonic insulator phase leads only to small changes in R_{xx} and R_{xy}. The formation of the excitonic insulator phase therefore significantly impacts only the component of the conductivity perpendicular to the planes, which is consistent with a density-wave periodic potential extending only along the c axis.

IV. DETERMINATION OF THE ACTIVATION GAP $E_{\rm a}$

Following a similar procedure as outlined by Fauqué *et al.* [3], we have evaluated the activation gap by making an Arrhenius plot. Here (in Fig. 6) we fit the function $R_{zz} = R_0(\exp(-E_{\rm a}/k_{\rm B}T) + r)^{-1}$ to the experimental interlayer resistivity data, where r represents a small proportion of the sample that remains conducting (presumably due to in-gap states) causing R_{zz} to saturate at low temperatures. The energy gap $E_{\rm a}$ gives rise to thermally activated transport. The increase in $E_{\rm a}$ at higher magnetic fields is evidenced by the increase in the slop of the Arrhenius plot.

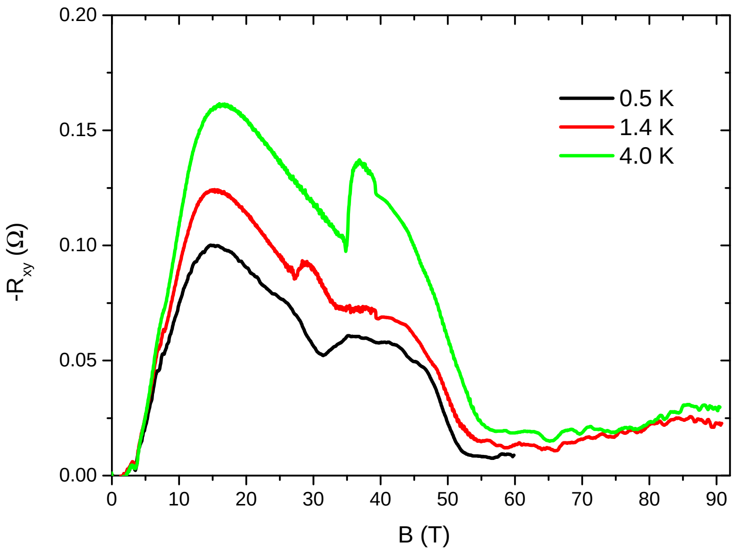

FIG. 5. The Hall resistace R_{xy} measured at 3 different temperatures in different colors, as indicated.

Under more general circumstances there are two energy gaps: $E_{\mathrm{a,min}} = \sqrt{E_{\mathrm{g}}^2 + \Delta^2}$ for the minority-spin Fermi surface and $E_{\mathrm{a,maj}} = \Delta$ for the majority-spin Fermi surface. The latter dominates owing to the more significant contribution of the minority-spins to the density of electronic states (i.e. $\mathrm{d}k_z/\mathrm{d}E$ is greater for the minority-spin bands, where here E is energy).

V. NEAR COINCIDENCE OF B_0 AND B_0'

While E_{a} continues to increase with field for $B > B_0$ in Fig. 3c of the main text, the interlayer magnetoresistance R_{zz} within the insulting regime at low temperatures reaches a maximum at a field $B_0' \approx 47$ T very close to B_0 (see Figs. 1b and 3b of the main text). This can be understood by noting that there are two different conductivity contributions to R_{zz} (see Fig. 6). The total R_{zz} is also governed by the low T saturation value R_0/r, which originates from in-gap states. The fact that r reaches an minimum near $B \approx B_0$ suggests that it originates primarily from majority-spin in-gap states. The gap on the majority-spin Fermi surface (see Figs. 2c and d of the main text) is determined by Δ (instead of $\sqrt{E_{\mathrm{g}}^2 + \Delta^2}$ as for the minority-spin bands), causing the number of majority-spin in-gap states and hence r/R_0 to be lowest when Δ is maximum at $B = B_0$.

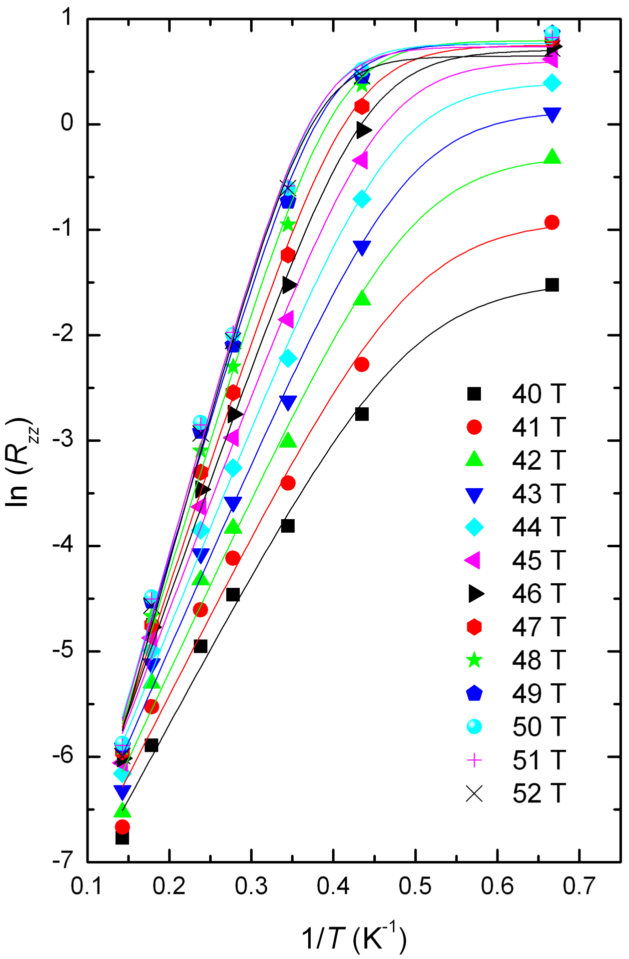

FIG. 6. R_{zz} versus $1/T$ at different fixed magnetic fields measured by Fauqué *et al.* [3], together with Arrhenius fits as described in the text.

VI. ANGLE-DEPENDENCE OF B_0

The values of B_0 in Figs. 3a and b of the main text are estimated from the field derivative of R_{zz} shown in Fig. 7. In the inset we show that on plotting $\partial R_{zz}/\partial B$ versus $B\cos\theta - B_0\cos\theta$ at each angle, the curves overlap, enabling us to ascertain that the maximum in $\partial R_{zz}/\partial B$ is reached at $B_0\cos\theta = 42.8$ T for $\theta = 47.6°$.

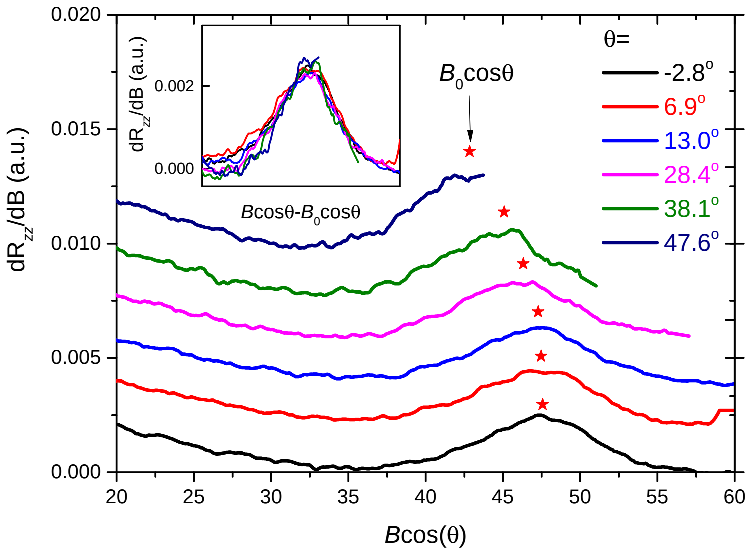

FIG. 7. $\partial R_{zz}/\partial B$ at 15K obtained from Fig. 3a of the main text at different angles θ as indicated. Red stars indicated the values of $B_0 \cos\theta$ at which the maximum is observed at each angle.

VII. TEMPERATURE-DEPENDENCE OF $E_{\rm g}$

The departure of the blue dotted line in Fig. 1b in the main text from being completely vertical (as well as the small difference in field between B_0' estimated from R_{zz} at 1.4 K and B_0 estimated from the inflection at 15 K in Fig. 2 of the main text) could also originate from a possible weak temperature dependence of $E_{\rm g}$. According to Halperin and Rice [4], a temperature dependence of the location of the band gap can be caused by changes in the relative equilibrium occupation of electron and hole states with temperature. Changes in occupation are more likely to occur if the electronic dispersions of the electron and hole bands are different. The weak temperature dependence of $E_{\rm g}$ in graphite suggests a small difference in the dispersion of the electron and hole states.

---

[1] A. A. Abrikosov, On the phase diagram of an excitonic insulator in a strong magnetic field, *Sov. Phys. JETP* **38**, 750-754 (1974).

[2] H. Yaguchi and J. Singleton, Destruction of the field-induced density-wave state in graphite by large magnetic fields, *Phys. Rev.Lett.* **81**, 5193-5196 (1998).

[3] B. Fauqué, D. LeBoeuf, B. Vignolle, M. Nardone, C. Proust, and K. Behnia, Two phase transitions induced by a magnetic field in graphite, *Phys. Rev. Lett.* **110**, 266601 (2013).

[4] B. I. Halperin, T. M. Rice, *Rev. Mod. Phys.* **40**, 755 (1968).
